# *Centella asiatica* Improves Physical Performance and Health-Related Quality of Life in Healthy Elderly Volunteer

**DOI:** 10.1093/ecam/nep177

**Published:** 2011-06-07

**Authors:** Lugkana Mato, Jintanaporn Wattanathorn, Supaporn Muchimapura, Terdthai Tongun, Nawanant Piyawatkul, Kwanchanok Yimtae, Panida Thanawirattananit, Bungorn Sripanidkulchai

**Affiliations:** ^1^Department of Biomedical Science Program, Graduate School, Khon Kaen University, 40002, Thailand; ^2^Department of Physiology, Faculty of Medicine, Khon Kaen University, 40002, Thailand; ^3^Department of Psychiatry, Faculty of Medicine, Khon Kaen University, 40002, Thailand; ^4^Department of Otolaryngology, Faculty of Medicine, Khon Kaen University, 40002, Thailand; ^5^Center for Research and Development of Herbal Health Product, Faculty of Pharmaceutical Sciences, Khon Kaen University 40002, Thailand

## Abstract

Recently, oxidative stress has been reported to contribute an important role in the decline of physical function as age advances. Numerous antioxidants can improve both physical and psychological performances resulting in the increase of health-related quality of life (HQOL). Therefore, we hypothesized that *Centella asiatica*, a medicinal plant reputed for nerve tonic, strength improvement and antioxidant activity, could improve the physical performance and HQOL especially in the physical satisfaction aspect, of the healthy elderly volunteer. To test this hypothesis, a double-blind, placebo-controlled, randomized trial was performed. Eighty healthy elderly were randomly assigned to receive placebo or standardized extract of *C. asiatica* at doses of 250, 500 and 750 mg once daily for 90 days. The subjects were evaluated to establish baseline data of physical performance using 30-s chair stand test, hand grip test and 6-min walk test. The health-related quality of life was assessed using SF-36. These assessments were repeated every month throughout the 3-month experimental period using the aforementioned parameters. Moreover, 1 month after the cessation of *C. asiatica* treatment, all subjects were also evaluated using these parameters again. The results showed that after 2 months of treatment, *C. asiatica* at doses of 500 and 750 mg per day increased lower extremity strength assessed via the 30-s chair stand test. In addition, the higher doses of *C. asiatica* could improve the life satisfaction subscale within the physical function subscale. Therefore, the results from this study appear to support the traditional reputation of *C. asiatica* on strength improvement, especially in the lower extremities of the elderly. *C. asiatica* also possesses the potential to be a natural resource for vigor and strength increase, in healthy elderly persons. However, further research is essential.

## 1. Introduction

As age advances, the functional fitness declines and results in poor quality of life [1, 2]. This situation has an enormous impact on the socio-economic status. The need for enhanced quality of life is increasing in its importance. Recent studies have shown the strong correlation between antioxidant consumption and physical performance in the elderly. Antioxidant consumption improved physical performance and physical strength [3]. Substances possessing antioxidant activity can have a positive effect on psychological stress [4]. Studies have also shown that the poor mental health score assessed using SF-36, and poor quality of life was associated with the low plasma level of antioxidants such as vitamin E [5]. Therefore, the development of food supplements to enhance the quality of life and physical performance of the elderly, has gained more concentration in recent times.

*Centella asiatica* (L) urban, a creeping plant in the family of Apiaceae, is found almost all over the world, including China, India and Thailand. This plant has been used in traditional folk healing for the ailments of various conditions including headache, body ache, insanity, asthma, leprosy, eczemas, and ulcers and wound healing [6, 7]. This plant also provides numerous beneficial effects for the nervous system such as anxiolytic [8] and anti-depression [9]. Recent studies both in animals and in human volunteers demonstrated that this medicinal plant could enhance cognitive function [10–13], as well as possessing antioxidant activity [14]. Moreover, it has been believed to enhance vigor and strength in the southern part of India.

Based on its reputation to improve both psychological and physical performances, and its antioxidant properties, the hypothesis that *C. asiatica* extract would have a positive effect on the physical strength and health-related quality of life, for the healthy elderly, was properly focused.

## 2. Methods

### 2.1. Participants

A total of 80, healthy, elderly volunteers, 4 male and 76 females (mean age 65.05 ± 3.56 years) were recruited to participate in this study. The Khon Kaen University, Faculty of Medicine, Ethics Committee, approved the study (ethical number HE490208). Inclusion criteria included healthy, elderly persons between the ages of 55 and 80, who were Thai Nationals dwelling in the Northeast Region of Thailand. Exclusion criteria included any history of cardiovascular diseases, respiratory diseases, neuropsychological diseases, head injury, diabetes, cancer, alcohol addiction, smokers of more than 10 pieces per day. Any persons taking prescribed and non-prescribed drugs or nutraceutical compounds influencing the function of the nervous system were also excluded.

Prior to participation, each volunteer signed an informed consent form and completed a medical health questionnaire. All recruited subjects were screened for medical health issues again, by the study physician, to assure the health status of all subjects prior to participation in the study. All subjects underwent an electrocardiogram, blood pressure and blood sugar evaluation.

All subjects also participated in an extensive medical evaluation process to ascertain subject suitability for entering the double-blind phase of the trial. All subjects were free of any herbal or prescribed medication that interfered or altered the function of the nervous system. Habitual smokers consuming more than 10 cigarettes per day were excluded from this study. All participants were requested and agreed to abstain from, caffeine containing products, throughout each study day, and alcohol for a minimum of 12 h prior to the test sessions.

### 2.2. Centella asiatica Preparation

A standardized aqueous extract of *C. asiatica* was prepared as described in patty patent (no. 4721, Thailand) by the Center for Research and Development of Herbal Health Product, Faculty of Pharmaceutical Sciences, Khon Kaen University. All *C. asiatica* used in this study was obtained from Tambon Sila, Khon Kaen Province. The plant was authenticated and kept as voucher specimen by the Faculty of Pharmaceutical Sciences, Khon Kaen University. Standardization and conformity of the extract was assured by strict in-process controls during manufacture and complete analytical control of the resulting dry extract. A-day capsule contained a specialized aerial part extract containing total phenolic content equivalent to tannic acid = 29.9 mg/g. In addition, the extract also contained asiaticoside and asiatic acid were presented at concentration of 1.09 and 48.89 mg/g of crude extract, respectively

### 2.3. Procedures and Treatments

This study was a pilot study conducted as a 12-week, double-blind, placebo-controlled, randomized trial. A random list of numbers was generated by computer. After being randomly assigned to various treatment groups, each participant received one capsule of placebo or *C. asiatica* extract at various doses of 250, 500 and 750 mg, once daily. Placebo and *C. asiatica* capsules had the same color, texture, size and smell.

This study assesses the physical performance consisting of strength and endurance. All participants were assessed for baseline data concerning physical fitness using a modified test of Rogers et al. [15] and Jones et al. [16]. The method of Rogers et al. assessed only the physical parameters associated with fall risk in older persons which consist of tests for balance assessment, walking velocity and muscle strength.

This study assessment consists of:

- Thirty-second chair stand test assessing lower extremity strength.
- Hand grip evaluating upper extremity strength.
- Six-minute walk test assessing aerobic endurance.

This study modified the method of Rogers et al. by using only the 30-s chair stand test and hand grip test to evaluate strength. This study also includes a 6-min walk test to assess endurance. All three of the tests that are used in this study are indicators of function and overall physical performance in the elderly.

Test subjects were assessed on their quality of life using the health quality of life questionnaire SF-36. Additional data pertaining to subjects social relationships, memory and learning were also collected. Subjects were assessed on their quality of life at a monthly interval, for the entire 3 month study. In addition, subjects were again assessed using the above mentioned parameters at 1 month post cessation of *C. asiatica* treatment. The code for study allocation was only broken when the last participant completed the entire follow up. Staff involved in the collection of the study's endpoints were instructed to follow a rigorous protocol and not to discuss any issues related to the use of medication. The review of compliance with medication and side-effects was performed independently by the investigators, who were also blinded to group allocation. Adverse effects were assessed during every study visit. Subjects were requested to call the study center if they experienced any medical problems during the 90-day study period.

### 2.4. Quality of Life Assessment

Health-related quality of life was assessed by SF-36 and additional information collected concerning subjects' social relationships, memory and learning. Evaluation of the SF-36 was done by an investigator who was blinded to the subjects. In brief, the SF-36 is a generic health survey designed to assess aspects of health that are not disease, treatment, or age specific. The SF-36 is a generic multidimensional instrument consisting of eight multi-item components representing physical functioning (PF; the extent to which health limits physical activities, such as self care, walking and climbing stairs); role functioning physical (RP; the extent to which physical health interferes with work or other daily activities); bodily pain (BP; the intensity of pain and the effect of pain on normal work, both inside and outside the home); general health perceptions (GH; personal evaluations of current health, health outlook and resistance to illness); vitality (VT; feeling full of energy rather than tired and worn out); social functioning (SF; the extent to which physical health or emotional problems interfere with normal social activities); role functioning emotional (RE; the extent to which emotional problems interfere with work or daily activities); mental health (MH; general mental health including depression, anxiety, behavioral-emotional control, and general positive affect) [17]. In this study, SF-36 scores in physical functioning aspects were converted to a scale of 0–100, a higher score indicating a better quality of life.

### 2.5. Statistical Analysis

All data are expressed as means ± SD. Comparisons between placebo and various doses of *C. asiatica* at different time points were made using repeated measurement analysis of variance (repeated-ANOVA). Statistical significance was set at *P*-value < .05.

## 3. Results

### 3.1. Characteristics of Subjects

Eighty elderly subjects participated in this study. The trial commenced with 76 female and 4 male volunteers. The entire study was conducted and completed with all 80 original study subjects. The baseline data concerning characteristics of subjects in all groups are shown in Table 1. No significant differences of parameters among various groups were observed.

### 3.2. Physical Fitness

The effects of *C. asiatica* on the 30-s chair stand test and 6-min walk test are shown in Table 2, with the effect of *C. asiatica* on the grip strength test shown in Table 3. It was found that *C. asiatica* extract at doses of 500 and 750 mg per day, showed improvement in the 30-s chair stand test results, significantly, at 2 months of treatment and the significance was still observed at 3 months of treatment (*F*(0.0500, 3.57) = 23.3794, *P* < .0001). When the treatment was prolonged to 3 months, the low dose of *C. asiatica* could also improve the 30-s chair stand test (*F*(0.0500, 3.57) = 5.2662, *P* = .0028) when compared with the baseline data. No time window of exposure dependent and dose dependent was observed on this parameter. The plant extract at all dose levels used in this study failed to show significant improvement on the 6-min walk test and hand strength test. No significant changes of these parameters were observed at 1 month post cessation of *C. asiatica* treatment for all dosage ranges used in this study. The data also showed that at 1 month post cessation of *C. asiatica* treatment, all significant changes disappeared.

### 3.3. Quality of Life

The effect of *C. asiatica* on life satisfaction was assessed using the QOL questionnaire and the data is shown in Table 4. After 2 months of *C. asiatica* treatment, significant improvement of the physical functioning subscale of SF-36 was observed (*F*(0.0500, 4.76) = 2.2741, *P* = .0690) when compared with the pre-dose score. However, 1 month post cessation of *C. asiatica*, no significant changes of physical domain of life satisfaction were observed.

## 4. Discussion

This study is the first study to show that *C. asiatica* can improve the performance in the 30-s chair stand test results, which is indicative of improvement in lower extremity strength. It was found that after the administration of *C. asiatica* extract at a period of 2 and 3 months, the performance of the elderly in 30-s chair stand test improved.

Previous study demonstrated that as the age advanced, the capacity of skeletal muscle to use oxygen decreased. In addition, the fiber diameter of skeletal muscle and its mass also decreased [18]. Abundant factors have been claimed to contribute to the important role of age-related skeletal muscle loss, including the increase of free radicals. Data obtained from animal study showed that the activity of glutathione peroxidase enzyme in skeletal muscle decreased [19] while the reactive oxygen species (ROS) increased due to the decrease of mitochondrial function [20]. *C. asiatica* was reported to improve oxidative stress [10].

**Figure 1:**
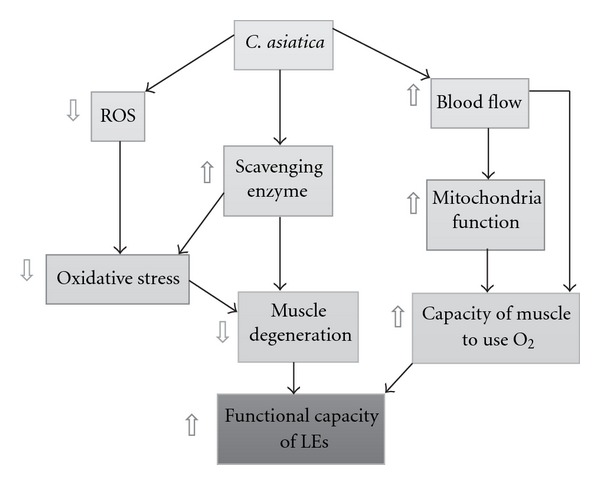
Effect of *C. asiatica* on the skeletal muscle of lower extremities. ROS: reactive oxygen species, O_2_: oxygen, LEs: lower extremities.

Recently, it was also shown that the increase of muscle blood flow played an important role on the metabolic implications, which in turn influenced the functional capacity of the muscle [21]. *C. asiatica* was also able to increase blood flow [22]. Taking all data together, we suggest that the possible underlying mechanism of *C. asiatica* to improve the muscle strength of lower extremities might occur partly via its antioxidant activity and partly via the increase of blood flow to skeletal muscle (Figure 1). However, the precise underlying mechanism will require further study.

It was shown that *C. asiatica* could improve only the strength of the muscle of lower extremities but not that of the upper extremities. The possible explanation for this phenomenon might be related to the differential distribution of type I and II skeletal muscle. It had been previously reported that muscle of the lower extremities appeared to contain more type I muscle fiber, which contained higher vascular supply, than the muscle of the upper extremities.

The health-related quality of life (HRQOL) in this study was assessed using the SF-36 questionnaire. Health is defined as a dynamic state of human wellbeing characterized by a physical, mental and social potential affected by “health” as defined above. HRQL has been introduced to assess people's health status. To date, a number of questionnaires have been developed to evaluate HRQL, and the 36-item Short Form Health Survey (SF-36) is the most commonly used. The SF-36 has been proven useful in monitoring population health, estimating the burdens of different diseases, monitoring outcome in clinical practice and evaluating medical treatment effects. It has been translated into many languages with its content examined cross cultures [23–26].

In addition, SF-36 has been used as an instrument for assessing quality of life world-wide. Normative data have also been obtained in many countries [27]. The SF-36 consists of eight multi-item components representing physical functioning, role functioning physical, bodily pain, general health perceptions, vitality, social functioning and role functioning emotional and mental health [17]. However, the main focus in this study was the physical functioning. The results from this study showed that *C. asiatica* improved the satisfaction score of physical fitness items. The improvement in physical fitness was observed only after 2 months of *C. asiatica* treatment at a high dose. Moreover, the significance was still observed when the treatment duration was increased further to 3 months. The changes observed using this tool were also correspondent with the alteration of physical activity. Therefore, this plant has a potential to improve physical fitness in healthy elderly.

The results obtained from this study failed to show the dose dependent manner. This might be related to the masking effect of other ingredients existing in the crude extract, of *C. asiatica* extract. Since the *C. asiatica* used in this study was the crude extract, the increased concentration of this plant extract might also increase the concentration of both active and non-active ingredients. Thus, the non-active ingredients might mask the effect of the active ingredient.

In conclusion, our study clearly demonstrated the potential of *C. asiatica* to improve muscle strength, especially the muscle of the lower extremities. *C. asiatica* might be useful as a natural resource in the development of functional foods improving the daily physical activity in the healthy elderly.

## Funding

The Development of Nutraceutical Compounds and Brain Plasticity Research Group, Faculty of Medicine and Center for Research and Development of Herbal Health Products, Khon Kaen University, Khon Kaen, Thailand.

## Figures and Tables

**Table 1:** Baseline characteristic of subjects.

Baseline data	Placebo	CA250	CA500	CA750
Age (years)	65.70 ± 4.88	64.60 ± 4.54	64.20 ± 5.11	66.75 ± 4.73 *F*(0.0500, 3.76) = 1.1380, *P* = .3392
Education (years)	10.90 ± 5.93	11.70 ± 5.55	11.25 ± 5.32	11.60 ± 6.08 *F*(0.0500, 3.76) = 0.0806, *P* = .9704
Blood pressure (mmHg)	120.75 ± 8.48/ 80.20 ± 6.35	116.40 ± 11.43/ 76.45 ± 7.04	119.90 ± 11.36/ 80.35 ± 7.62	121.80 ± 9.99 *F*(0.0500, 3.76) = 1.0165, *P* = .3902
				79.20 ± 6.12 *F*(0.0500, 3.76) = 1.4091, *P* = .2467
Blood sugar	94.75 ± 12.49	92.45 ± 5.89	93.20 ± 7.32	95.20 ± 12.40 *F*(0.0500, 3.76) = 0.3351, *P* = .8000
Body mass index	24.54 ± 4.33	22.87 ± 3.24	23.25 ± 2.50	23.75 ± 3.03 *F*(0.0500, 3.76) = 0.9312, *P* = .4299

Subjects were measured baseline characteristic. Data are presented as mean ± SD. *P*- and *F*-values were compared between groups (*n* = 20/group).

**Table 2:** Effect of C. asiatica on physical fitness measured by 30-s chair stand test and 6-min walk test.

Items	Pre-dose baseline score		Post-dose score			
			1 month	2 month	3 month	Delay
30-s chair stand test (times)					16.05 ± 2.72	16.10 ± 3.67
	Placebo	15.85 ± 2.87	15.65 ± 2.80	15.70 ± 2.99	*F*(0.0500, 3.57) = 0.3352,	*F*(0.0500, 1.19) = 0.1351,
					*P* = .7999	*P* = .7172
					20.35 ± 4.82^a,∗∗^	18.60 ± 5.96
	CA250	16.85 ± 4.21	18.40 ± 4.07	18.35 ± 4.85	*F*(0.0500, 3.57) = 5.2662,	*F*(0.0500, 1.19) = 2.2391,
					*P* = .0028	*P* = .1510
					20.35 ± 3.66^b,∗∗^	17.30 ± 4.03
	CA500	16.80 ± 4.36	17.75 ± 3.80	19.90 ± 4.10^a,∗∗^	*F*(0.0500, 3.57) = 23.3794,	*F*(0.0500, 1.19) = 1.3768,
					*P* < .0001	*P* = .2551
					21.00 ± 4.48^b,∗∗∗^	17.90 ± 5.93
	CA750	16.80 ± 3.09	17.45 ± 3.53	18.55 ± 3.17^a,∗^	*F*(0.0500, 3.57) = 13.9752,	*F*(0.0500, 1.19) = 0.9200,
					*P* < .0001	*P* = .3495

6 min walk test (meters)					518.35 ± 59.62	510.55 ± 78.06
	Placebo	515.60 ± 42.18	509.45 ± 55.78	504.95 ± 57.32	*F*(0.0500, 3.57) = 1.0645,	*F*(0.0500, 1.19) = 0.1759,
					*P* = .3714	*P* = .6796
					535.60 ± 52.40	519.25 ± 81.86
	CA250	517.60 ± 59.75	527.95 ± 42.34	524.25 ± 49.77	*F*(0.0500, 3.57) = 1.6551,	*F*(0.0500, 1.19) = 0.0155,
					*P* = .1869	*P* = .9022
					521.35 ± 66.20	512.00 ± 67.95
	CA500	509.50 ± 57.38	517.00 ± 64.62	523.10 ± 67.8	*F*(0.0500, 3.57) = 1.8655,	*F*(0.0500, 1.19) = 0.0355,
					*P* = .1457	*P* = .8526
					524.75 ± 80.68	520.55 ± 87.65
	CA750	511.75 ± 54.12	517.80 ± 75.67	529.05 ± 82.27	*F*(0.0500, 3.57) = 1.2784,	*F*(0.0500, 1.19) = 0.5046,
					*P* = .2905	*P* = .4861

Subjects were measured physical fitness measured by 30-s chair stand test and 6-min walk test. Data are presented as mean ± SD. *P*- and *F*-values were compared between pre-dose baseline score and post-dose 1, 2, 3 month and 1 month after cessation of treatment (*n* = 20/group).^a, b^ *P*-value <.01 and.001 compared with pre-dose baseline score, respectively.

*, **, ***P-value <.05,  .01 and  .001 compared with placebo treated group, respectively.

**Table 3:** Effect of *C. asiatica* on physical fitness measured by hand grip.

Hand strength	Pre-dose baseline score		Post-dose score			
			1 month	2 month	3 month	Delay
Left side (kg)					23.65 ± 3.93	23.85 ± 4.33
	Placebo	23.05 ± 4.69	22.95 ± 4.57	23.48 ± 4.46	*F*(0.0500, 3.57) = 0.6867,	*F*(0.0500, 1.19) = 3.5145,
					*P* = .563	*P* = .0763
					23.03 ± 6.19	22.20 ± 3.11
	CA250	21.55 ± 3.45	22.28 ± 2.96	22.70 ± 2.34	*F*(0.0500, 3.57) = 1.5077,	*F*(0.0500, 1.19) = 2.8391,
					*P* = .2223	*P* = .1084
						23.25 ± 4.98
					23.93 ± 5.64	*F*(0.0500, 1.19) = 1.6032,
	CA500	24.80 ± 7.88	23.90 ± 6.59	22.98 ± 5.30	*F*(0.0500, 3.57) = 1.3040,	*P* = .2208
					*P* = .2820	24.80 ± 6.17
					25.75 ± 6.45	*F*(0.0500, 1.19) = 0.0293,
	CA750	24.85 ± 6.29	25.50 ± 6.55	26.15 ± 7.30	*F*(0.0500, 3.57) = 2.2607,	*P* = .8660
					*P* = .0911	

Right side (kg)					24.15 ± 5.09	23.95 ± 5.31
	Placebo	23.43 ± 5.63	23.33 ± 5.24	24.05 ± 5.44	*F*(0.0500, 3.57) = 1.3691,	*F*(0.0500, 1.19) = 2.1713,
					*P* = .2614	*P* = .1570
						23.35 ± 2.50
					23.30 ± 3.11	*F*(0.0500, 1.19) = 0.0381,
	CA250	23.40 ± 2.67	24.05 ± 3.78	23.38 ± 3.01	*F*(0.0500, 3.57) = 1.2931,	*P* = .8474
					*P* = .2856	24.00 ± 5.99
					25.93 ± 6.09	*F*(0.0500, 1.19) = 3.5531,
	CA500	26.58 ± 7.95	24.7 ± 6.32	24.38 ± 5.16	*F*(0.0500, 3.57) = 2.0774,	*P* = .0748
					*P* = .1133	25.25 ± 6.34
					25.45 ± 5.27	*F*(0.0500, 1.19) = 2.8239,
	CA750	26.18 ± 5.53	26.40 ± 5.44	26.98 ± 5.41	*F*(0.0500, 3.57) = 2.0055,	*P* = .1092
					*P* = .0617	

Subjects were measured physical fitness by hand grip. Data are presented as mean ± SD. *P*- and *F*-values were compared between pre-dose baseline score and post-dose 1, 2, 3 month and 1 month after cessation of treatment (*n* = 20/group).

**Table 4:** Effect of various doses of *C. asiatica* on the satisfaction physical fitness score which assessed using QOL questionnaire in healthy elderly volunteer.

Aspect	Pre-dose baseline score		Post-dose			
			1 month	2 month	3 month	Delay
Physical Functioning						80.00 ± 13.28
	Placebo	84.50 ± 11.96	82.00 ± 13.07	80.13 ± 9.68	79.00 ± 9.26	*F*(0.0500, 4.76) = 2.3261,
						*P* = .0639
						80.50 ± 15.25
	CA250	78.25 ± 14.60	77.63 ± 16.13	80.75 ± 10.26	78.75 ± 12.13	*F*(0.0500, 4.76) = 1.0113,
						*P* = .4070
						81.63 ± 13.01
	CA500	84.13 ± 11.59	82.88 ± 10.74	84.63 ± 10.86	86.00 ± 7.75*	*F*(0.0500, 4.76) = 2.2900,
						*P* = .0674
						80.50 ± 10.66
	CA750	78.89 ± 9.46	80.4 ± 9.9	82.88 ± 7.79^b^	83.38 ± 7.53^a^	*F*(0.0500, 4.76) = 2.2741,
						*P* = .0690

Subjects were measured the satisfaction score which assessed using QOL questionnaire. Data are presented as mean ± SD. *P*- and *F*-values were compared between pre-dose baseline score and post-dose score for 1, 2, 3 month and 1 month after the cessation of *C. asiatica* treatment (*n* = 20/group).

^a, b^ *P*-value < .05 and .001 compared with pre-dose baseline score, respectively.

*P-value < .05 compared with placebo treated group.
